# Health literacy and blood glucose among Guyanese emergency department patients without diagnosed diabetes: a cross-sectional study

**DOI:** 10.1186/s13098-015-0028-1

**Published:** 2015-04-08

**Authors:** Candace D McNaughton, Rosalynne R Korman, Edmond K Kabagambe, Seth W Wright

**Affiliations:** Vanderbilt University, 703 Oxford House, 1313 21st Avenue South, Nashville, TN USA; Vanderbilt University, Suite 334J, 2525 West End Ave, Nashville, TN USA

**Keywords:** Health literacy, Blood glucose, Emergency department, Glycated hemoglobin

## Abstract

**Background:**

Low health literacy is associated with worse glycemic control among patients with diabetes; the relationship between health literacy and blood glucose among patients without diagnosed diabetes, particularly in resource-limited settings, is not known. Because emergency department patients are at risk for both low health literacy and undiagnosed diabetes, we examined their relationships among emergency department patients at the Georgetown Public Hospital Corporation in Guyana.

**Methods:**

We conducted a cross-sectional study across random time blocks from May to August 2012 among Guyanese emergency department patients without a diagnosis of diabetes. Health literacy was assessed by the Single Item Literacy Screener (SILS, range 1-5); low health literacy was defined as SILS ≥ 3. We examined the relationships among health literacy, random blood glucose (RBG), and point-of-care glycated hemoglobin (HbA1c).

**Results:**

Of the 228 enrolled patients, 125 (54%) were female, median age was 43 years (interquartile range 38 to 53), mean body mass index (BMI) was 25.6 kg/m^2^ (standard deviation 6.8 kg/m^2^), and 103 (45.2%) had low health literacy. The receiver operating characteristic area under the curve for RBG to detect elevated HbA1c (≥48mmol/mol) was 0.94 (95% CI: 0.91-0.97). After adjustment for age, sex, BMI, ethnicity, and education, the odds of having HbA1c ≥ 48 mmol/mol, consistent with undiagnosed diabetes, rose with decreasing health literacy (OR 2.2, 95% CI 1.2-3.8, p = 0.007, per point decrease in literacy).

**Conclusion:**

This pilot study of Guyanese emergency department patients without diagnosed diabetes found that low health literacy was common and was associated with higher HbA1c and random blood glucose.

## Background

Patients who seek care in the emergency department are at high risk for undiagnosed diabetes [1]; in one study, 2.8% of adult emergency department patients met criteria for undiagnosed diabetes [2,3]. Emergency department patients often have less access to preventative care [4], which may contribute to their higher risk for undiagnosed diabetes. Health literacy is the ability to obtain, read, understand, and use healthcare information to make appropriate health decisions and following instructions for treatment [5]. Poor health literacy, which is the ability to read and understand health information at lower than the 5^th^ grade level, is common and has been associated with less primary care use, worse clinical outcomes, and increased health care costs [6-10]. The prevalence of low health literacy among emergency department patients is high, between 25% and 74% in the United State [11,12]; the prevalence among patients in low- and middle-income countries is not known. Low health literacy has been tied to poor glycemic control among patients with diabetes in industrialized countries [13-16], but the relationship between health literacy and blood glucose levels among patients without diagnosed diabetes, particularly in low- and middle-income countries, is not known. Health literacy has also not been extensively studied in low resource settings, particularly in acute care settings such as the emergency department, although it has been assumed that the inverse relationship between health literacy and blood glucose found in industrialized countries exists in resource limited setting as well [17].

Diabetes is highly prevalent in the Caribbean countries, and prevalence is expected to rise as the population becomes more obese at younger ages [18-22]. Guyana is part of the community of Caribbean countries and is thought to have a similarly high prevalence of diabetes [23-26]. Guyana has an adult diabetes prevalence of 15.9%, the second highest prevalence of any country in the Americas. For example, diabetic foot disease has become so common that the primary Guyanese referral hospital now runs a clinic specializing in diabetic foot ulcer evaluation and treatment, and satellite diabetic foot clinics have been established in some regional hospitals and health centers.

To our knowledge, no prior work has evaluated the relationship between health literacy and undiagnosed diabetes among emergency department patients in low- and middle-income countries. Therefore, using a cross-sectional convenience sample of patients without a prior history of diabetes who sought medical care in an emergency department in Guyana, we explored the relationship between patient-reported health literacy and blood glucose, as measured by random blood glucose (RBG) and point-of-care (POC) testing of glycated A1c (HbA1c), hypothesizing that low health literacy would be associated with higher RBG and HbA1c. Because POC HbA1c testing is not available in many emergency departments in developing countries, we also evaluated whether POC HbA1c could be estimated from the readily available RBG.

## Methods

From May 21, 2012 to August 7, 2012, we conducted a cross-sectional study of patients who sought medical care at the Georgetown Public Hospital Corporation (GPHC) emergency department in Georgetown, Co-operative Republic of Guyana. Guyana is an English-speaking former British colony located on the northern coast of South America. The majority of the population lives in the capital city of Georgetown and along a narrow coastal strip; the interior jungle and savannah regions are sparsely populated. Guyana is ethnically diverse with most of the population of South Asian, African and/or Amerindian heritage. Guyana is culturally and economically tied to the Caribbean nations and is classified as a Caribbean country by the International Diabetes Federation [26]. Guyana has a lower-middle-income economy and is in a state of epidemiological transition [27,28]. The Guyana Ministry of Health (MOH) coordinates a comprehensive medical care system of regional and district hospitals, community health centers, and rural health posts. GPHC is a semi-autonomous public teaching hospital that is the major tertiary care center for the country and serves a diverse socioeconomic population. Medical care, including essential medications for diabetes, is provided without charge at all MOH facilities and at GPHC. The emergency department at GPHC sees 90,000 patients per year and is staffed by residents from the University of Guyana/GPHC residency program and by general medical officers.

Non-pregnant patients aged 30 or older with no history of diabetes who presented to the emergency department were eligible for the study. Age 30 was chosen as a cutoff based on work by Kahn et al., which found that age 30 was a cost effective threshold to initiate diabetes screening [29]. GPHC has a three level triage system. Patients triaged as level 1 (emergent) were excluded from the study. Patients triaged as level 2 (urgent) or level 3 (non-urgent) who were considered by the research assistants to be medically or psychologically unstable for participation in the study (e.g. sexual assault, severe pain, heavily intoxicated, active bleeding) were excluded. Those referred for a high blood sugar and those receiving glucose containing intravenous fluids on arrival were also excluded. Patients were approached for enrollment on a convenience basis during daytime hours on weekdays and weekends when the investigators were available. Trained research assistants administered the health literacy survey and collected demographic information and diabetes risk factors. Participants provided fingerstick samples of blood for RBG (LifeFirst OneTouch**®**, Lifescan, Inc., Milpitas, CA) and HbA1c (Siemens DCA Vantage™ Analyzer, Siemens Healthcare Diagnostics, Inc., Tarrytown, NY). The POC HbA1c is based on the latex agglutination inhibition immunoassay method and provides results in 6 minutes. It was chosen specifically because it meets accepted analytical performance criteria, with a coefficient of variation <3% in the clinically relevant range, and its performance is not significantly impacted by the presence of hemoglobin S or C; it does not, however, provide a reading if overall hemoglobin is <4.3 mmol/l (<7 mg/dL) [30,31]. Data collection and testing was completed in approximately 15 to 20 minutes. Written informed consent was obtained from all participants. The institutional review boards of both Vanderbilt University Medical Center and the Georgetown Public Hospital Corporation approved this study.

### Exposure

Patient-reported health literacy was assessed using the Single Item Literacy Screener (SILS), which was designed to identify patients who need help reading and understanding health information [32]. The SILS was chosen because prior work has shown that patients prefer self-reported measures of health literacy [33,34], and the time constraints of the emergency department setting preclude use of longer, content-based measures. The SILS has been used among general outpatient clinic and ED adult patients in other English-speaking countries [32,33,35]. Participants were asked to answer the single question “How often do you need to have someone help you when you read instructions, pamphlets, or other written material from your doctor or pharmacy?” on a 5-point Likert-like scale (1-Never, 2-Rarely, 3-Sometimes, 4-Often, and 5-Always); a higher SILS score indicates lower health literacy. Low health literacy was defined as an answer of “Sometimes,” “Often,” or “Always” (SILS ≥ 3) based on prior work that showed this threshold to have 83% specificity (95% confidence interval [CI] 81%-86%) and 54% sensitivity [95% CI: 47% - 61%] for detecting low health literacy [32].

### Dependent variables

Measurement of blood glucose by RBG and HbA1c occurred at approximately the same time. While prior work has evaluated the relationship between RBG and HbA1c among fasting patients in clinic settings, to our knowledge this has not been studied in the emergency department setting. HbA1c was recorded as percent hemoglobin and converted to the international system of units (SI) with the following equation: (HbA1c*10.93)-23.50. RBG was recorded in mg/dL and converted to SI with the following equation: RBG/18. Our dependent variable, elevated HbA1c, was defined as 1 if HbA1c was ≥48 mmol/mol (or 6.5%) and 0 if it was less. This 48 mmol/mol is the threshold used to define diabetes by the American Diabetes Association and Caribbean Health Research Council [36,37].

### Statistical analysis

Demographics and clinical characteristics are presented as frequencies and means with standard deviations (sd), as appropriate. The independent variable each model was health literacy as measured by the SILS, with a higher score indicating lower health literacy. We used logistic regression models to study the associations between RBG and HbA1c in models adjusted for age, gender, body mass index (BMI), ethnicity and education level, as in previous studies [13,15,38-40]. Less than 5% of data was missing; complete case analyses were performed. Analyses were conducted using Stata 11.2 (StataCorp LP, College Station, TX). Model fitness was checked using the Hosmer-Lemeshow test. Associations were considered significant at *P* < 0.05.

In order to determine whether RBG can be used to detect HbA1c, we fitted a logistic regression model with covariates stated above and created a receiver operating characteristic and estimated the area under the curve (ROC AUC) with its corresponding 95% CI, as well as the sensitivity and specificity at each RBG value.

## Results

A total of 1,010 patients presented during study time blocks; of these, 270 met initial inclusion criteria and were approached for potential consent, and 230 (85%) consented to participate. One subject did not complete the SILS, one participant did not undergo HbA1c testing for technical reasons, and seven were too anemic (hemoglobin <7 mg/dL, or 4.3 mmol/l) to accurately measure HbA1c. Thus, 228 patients were included in the analysis evaluating the relationship between health literacy and RBG, and 220 participants were included in the analysis of the relationships among health literacy, RBG, and HbA1c.

**Figure 1:**
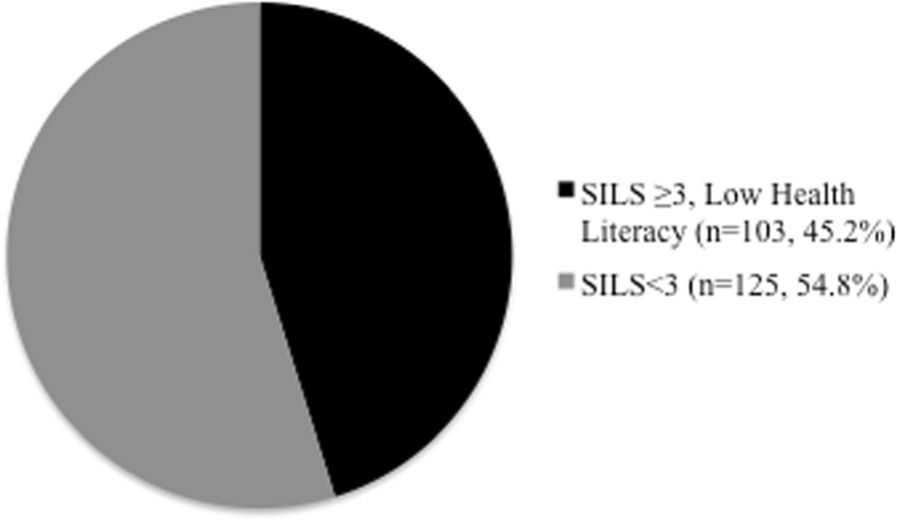
**Heath Literacy categorized by the Single Item Literacy Screen (SILS).**

Clinical characteristics, stratified by health literacy level, are found in Table 1. Of 228 participants, 125 (54%) were female, median age was 43 years (interquartile range [IQR] 38 to 53), and 103 (45%) had low health literacy (Figure 1). By self-report, 90 (39%) participants reported that they had previously been tested for diabetes, and 87 (38%) reported a first-degree family history of diabetes. Personal past medical history included hypertension for 50 participants (22%), hyperlipidemia for 31 (14%), and heart disease or stroke for 11 (5%), while 198 (97%) reported they were physically active.

**Table 1:** **Patient characteristics (n = 228)**

**Characteristics**	**SILS ≥ 3**	**SILS < 3**	**p-value**
	**(Low health Literacy n = 103)**	**n = 125**	
Age in years, mean (sd)	46.2 (11.5)	46.7 (12.5)	0.78**
Female, no. (%)	65 (52.0)	60 (57.1)	0.34*
Ethnicity, no. (%)			
Afro-Guyanese	38 (36.9)	59 (47.2)	0.85***
Amerindian	7 (6.8)	3 (2.4)	
Indo-Guyanese	38 (36.9)	26 (20.8)	
Mixed	20 (19.4)	37 (29.6)	
Education level no. (%)			
None	9 (8.7)	3 (2.4)	<0.001***
Primary	50 (48.5)	33 (26.8)	
Secondary	41 (39.8)	67 (54.5)	
Tertiary	3 (2.9)	20 (16.3)	
BMI, mean (sd)	25.2 (6.2)	25.9 (7.3)	0.80**
Prior testing for DM, no. (%)	39 (37.9)	51 (40.8)	0.65*
Family history of DM, no. (%)	43 (41.8)	44 (35.2)	0.31*
Reported history of HTN, no. (%)	22 (21.4)	28 (22.4)	0.85*
Hyperlipidemia, no. (%)	16 (15.5)	15 (12.0)	0.43*
Physically active, no. (%)	90 (87.4)	108 (86.4)	0.83*
RBG, mmol/l, mean (sd)	7.12 (3.5)	6.5 (2.1)	0.20**
	**N = 98**	**N = 122**	
HbA1c, mmol/mol (sd)	39.1 (8.1)	37.9 (7.3)	0.43**
HbA1c ≥48 mmol, no. (%)	7 (7.1)	2 (1.6)	0.04*

*Abbreviations:* SILS, single item literacy screener; sd, standard deviation; no, number; BMI, body mass index; DM, diabetes mellitus; HTN, hypertension.

*by Chi squared test;**by Kruskall-Wallis test; ***by nonparametric test for trend.

Table 1 presents RBG and HbA1c stratified by health literacy level, and Table 2 presents results of logistic and linear regressions evaluating the relationship between health literacy (continuous; higher SILS indicates lower health literacy) and blood glucose measured by RBG and HbA1c, as well as elevated HbA1c (HbA1c ≥ 48 mmol/mol). After adjusting for age, sex, body mass index, ethnicity, and education level, the adjusted odds of having HbA1c ≥48 mmol/mol, consistent with undiagnosed diabetes, rose with decreasing health literacy: OR 2.2 (95% CI 1.2 to 3.8; p = 0.007) for each point increase in the SILS. Overall, lower patient-reported health literacy was associated with higher blood glucose as measured by RBG and HbA1c, although 95% CI crossed the null.

**Table 2:** **Relationships of health literacy with random blood glucose and HbA1c**

**Logistic regression**	**Unadjusted OR (95% CI)**	Adjusted model OR (95% CI)**
HbA1c ≥ 48mmol	2.0 (1.2 to 3.3), p = 0.005	2.2 (1.2 to 3.8), p = 0.007
**Linear regression**	**Unadjusted Beta (95% CI)**	Adjusted Model Beta (95% CI)*
RBG, mmol/l	0.28 (0.04, 0.53), p = 0.02	0.20 (-0.05, 0.46), p = 0.12
HbA1c, mmol/mol	0.61 (-0.07, 1.28), p = 0.08	0.66 (-0.04, 1.36), p = 0.07

* per point increase in the Single Item Literacy Screener (SILS), where higher SILS indicates lower health literacy ; **adjusted for age, gender, BMI (body mass index, kg/m^2^), ethnicity, and education.

Abbreviations: 95% CI, 95% confidence interval; HbA1c, glycated hemoglobin A1c; RBG, random blood glucose; sd, standard deviation; no., number; OR, odds ratio.

**Figure 2:**
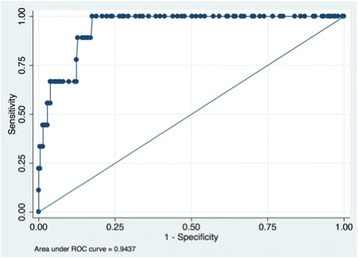
**ROC AUC for RBG detecting HbA1c ≥ 48mmol/mol: 0.94 (95% CI 0.91-0.97).** Abbreviations: ROC AUC, receive operating characteristics area under the curve; HbA1c, glycated hemoglobin; CI, confidence interval.

Mean SILS score was 3.7 (sd 1.5) out of a maximum score of 5, and 103 (45%) had low health literacy (SILS ≥ 3). Mean RBG was 6.8 mmol/l (sd 2.9 mmol/L), and mean HbA1c was 38.4 mmol/mol (sd 7.7 mmol/mol). Forty-seven (20.6%) of the participants reported no caloric intake within the prior eight hours, and an additional 127 (55.7%) reported no caloric intake within the prior 4 hours. The ROC curve for RBG to detect elevated HbA1c is found in Figure 2. RBG ≥ 7.2 mmol/L (129 mg/dl) was 100% sensitive and 79% specific for HbA1c ≥ 48 mmol/mol. RBG ≥ 8.6 mmol/l (154 mg/dL) was 67% sensitive and 92% specific for HbA1c ≥ 48 mmol/mol.

## Discussion

In this cross sectional study of 228 adult patients without known diabetes who sought medical care in the emergency department of a large urban hospital in Guyana, we found that low health literacy was common and that lower patient-reported health literacy was associated with higher blood glucose (Table 2). We also found that RBG has reasonable test characteristics for identifying elevated HbA1c (ROC AUC = 0.94, 95% CI 0.91-0.97).

To our knowledge, this is the first study to explore the relationship between patient-reported health literacy and blood glucose in a low- or middle-income country. Low health literacy was common in this patient population, with 103 patients (45.2%) reporting that they “sometimes,” “often,” or “always” “…need to have someone help…when [they] read instructions, pamphlets, or other written material from [their] doctor or pharmacy” [32]. General education is only loosely associated with health literacy (correlation between the two in this data was only 0.33), therefore the ability to read and understand general materials does not necessarily translate into the ability to use and understand medical literature or materials. As a result, highly educated patients sometimes have very low health literacy levels. In order to examine the relationship between health literacy and glucose separate from general education level, our analyses adjusted for education level.

Low health literacy among patients with diagnosed diabetes may be associated with elevated blood glucose due to poor diet and lifestyles choices, difficulty performing disease self-management tasks, or poor medication adherence [41-43]. The relationship between health literacy and blood glucose has not been studied among patients without diagnosed diabetes, but we hypothesized that low health literacy may be associated with higher blood glucose if health literacy results in poor health behaviors, unawareness of symptoms of diabetes, or barriers to accessing the health care system to receive diagnosis of and treatment for diabetes. Because the SILS is more specific than sensitive for detecting low health literacy, our results may underestimate the true burden of low health literacy among Guyanese emergency department patients, although the proportion of patients with low health literacy in our study is similar to the proportion of patients with low health literacy in lower socioeconomic areas of the United States, where the prevalence of low health literacy varies between 25% and 74% [11,12,44].

Annually more than 2.8% of emergency department patients likely have undiagnosed diabetes [1], potentially due to decreased access to care [45] and/or decreased understanding of and adherence to lifestyle changes and medical therapy. This study was conducted because clinical experience suggested that Guyanese emergency department patients were at risk for undiagnosed diabetes; we also sought to understand patient characteristics that may be related to undiagnosed diabetes. We hypothesized that among emergency department patients in Guyana, higher patient-reported health literacy would be associated with lower blood glucose. This hypothesis implies that a portion of these patients has undiagnosed diabetes and another portion will go on to develop diabetes in the future. Therefore, having a clear understanding of the relationship between patient-reported health literacy and blood glucose may help to inform future interventions targeting particularly high risk populations such as emergency department patients for more aggressive diabetes screening, lifestyle interventions, and treatment.

Because diagnosing diabetes generally requires confirmatory testing that is not generally available in the emergency department setting, we used RBG combined with POC HbA1c testing and encouraged patients with abnormal values to follow up with a primary care provider for additional testing [36]. Our results indicate that for each point decrease of the SILS, the odds of having HbA1c ≥ 48 mmol/mol rose, with an adjusted OR of 2.2 (95% CI 1.2 to 3.8, p = 0.007). Surprisingly, we found that RBG was sensitive and specific for elevated HbA1c, with a c-statistic of 0.94 (95% CI 091, 0.97), similar to that of fasting glucose levels among clinic patients. Prior work has shown that emergency department patients who have RBG ≥ 8.6 mmol/l in the presence of symptoms of diabetes including polyuria, polydipsia, fatigue, or weight loss were all later confirmed to have diabetes or prediabetes [46]. In our study, 47 (20.6%) of patients reported they had fasted for the 8 hours prior to blood glucose testing; of those who had eaten in the prior 8 hours, mean time since caloric intake was 3.4 hours (sd 2.0). These findings are preliminary but lend support to expert recommendations that under some conditions close follow-up may be considered for patients with very high blood glucose in the emergency department.

This study should be interpreted within the following limitations. GPHC is the only tertiary care referral hospital in Guyana and patients who seek care there may be fundamentally different from patients who seek care in more rural areas of Guyana or in other countries. We used POC HbA1c testing, which may be less reliable than laboratory based high-performance liquid chromatography methods; the DCA Vantage^TM^ Analyzer was chosen specifically for its reliability and because it is unaffected by the presence of hemoglobin S and C variants. Using HbA1c ≥ 48 mmol/mol (6.5%) to define elevated HbA1c, only two patients with elevated HbA1c had high literacy. This small sample size limits our power to fully explore the relationship between health literacy with RBG and HbA1c and may be at risk for instability. Prior history of diabetes and testing for diabetes were by patient report; it is possible that patients with low literacy may have been diagnosed with diabetes but they were unaware of the diagnosis. Lastly, the SILS measures health literacy by asking patients about how often they need help reading hospital materials. Patient responses may be influenced by social desirability bias and inaccurate patient perception, although the SILS has been successfully validated and used in multiple settings [32,33,35], and its use permits rapid identification of patients with low health literacy in the emergency department setting.

In conclusion, this pilot cross-sectional study of 228 Guyanese emergency department patients without known diabetes found that lower patient-reported health literacy was associated with elevated HbA1c, and the c-statistic for random blood glucose and HbA1c ≥ 48 mmol/mol was 0.94 (95% CI 0.91-0.97). Given the high burden of low health literacy and diabetes in Guyana and other low- and middle-income countries, future work will be important to confirm these findings and more fully explore potential mechanisms by which health literacy may be related to blood glucose among patients without diagnosed diabetes.

## Abbreviations

BMI: Body mass index
DM: Diabetes mellitus
GPHC: Georgetown Public Hospital Corporation
HbA1c: Testing of glycated A1c
HTN: Hypertension
MOH: Ministry of health
POC: Point-of-care
RBG: Random blood glucose
ROC AUC: Receiver operating characteristic area under the curve
SI: International system of units
SILS: Single Item Literacy Screener
